# Effects of multicomponent training and HMB supplementation on disability, cognitive and physical function in institutionalized older adults aged over 70 years: a cluster-randomized controlled trial

**DOI:** 10.1016/j.jnha.2024.100208

**Published:** 2024-03-14

**Authors:** Héctor Gutiérrez-Reguero, Ángel Buendía-Romero, Francisco Franco-López, Alejandro Martínez-Cava, Alejandro Hernández-Belmonte, Javier Courel-Ibáñez, Ignacio Ara, Julian Alcazar, Jesús G. Pallarés

**Affiliations:** a GENUD Toledo Research Group, Faculty of Sports Sciences, University of Castilla-La Mancha, Toledo, Spain; b CIBER on Frailty and Healthy Aging (CIBERFES), Instituto de Salud Carlos III, Madrid, Spain; c Instituto de Investigación Sanitaria de Castilla-La Mancha (IDISCAM), Junta de Comunidades de Castilla-La Mancha (JCCM), Spain; d Human Performance and Sports Science Laboratory, Faculty of Sport Sciences, University of Murcia, Murcia, Spain; e Department of Physical Education and Sport, University of Granada, Granada, Spain

**Keywords:** Aging, Lower-limb power, Beta-hydroxy-beta-methylbutyrate, Exercise training, Nursing home

## Abstract

**Objectives:**

To investigate the synergist effects of exercise and β-hydroxy β-methylbutyrate (HMB) supplementation on disability, cognitive and physical function, and muscle power in institutionalized older people.

**Design:**

Cluster-randomized controlled trial.

**Participants:**

Seventy-two institutionalized older adults (age = 83 ± 10 years old; 63% women) were randomized in four groups: exercise plus placebo (EX), HMB supplementation, EX plus HMB supplementation (EX + HMB), and control (CT).

**Intervention:**

The exercising participants completed a 12-week tailored multicomponent exercise intervention (Vivifrail; 5 days/week of an individualized resistance, cardiovascular, balance and flexibility program), whereas the HMB groups received a drink containing 3 g/day of HMB.

**Measurements:**

Participants were assessed Pre and Post intervention for disability and cognitive function (validated questionnaires), physical function (short physical performance battery, SPPB), handgrip strength and sit-to-stand relative muscle power. Linear mixed-effect models were used to compare changes among groups.

**Results:**

Compared to baseline, both EX and EX + HMB improved cognitive function (+2.9 and +1.9 points; p < 0.001), SPPB score (+2.9 points and +2.4 points; p < 0.001) and relative muscle power (+0.64 and +0.48 W·kg^−1^; p < 0.001), while CT and HMB remained unchanged (p > 0.05). Significant between-group differences were noted between CT, EX and EX + HMB for cognitive function (p < 0.01), between CT and EX + HMB for physical function (p = 0.043), and between CT, EX and EX + HMB for relative muscle power (p < 0.001).

**Conclusion:**

The Vivifrail exercise program was effective in improving cognitive and physical function, and muscle power in nursing home residents, while HMB supplementation did not provide additional benefits when combined with exercise. These results emphasize the importance of physical exercise interventions in very old people as an essential basis for improving their overall health and quality of life.

## 1 Introduction

Nowadays, thanks to technological advances and improvements in healthcare, life expectancy has increased worldwide by approximately 6 years in the last 20 years [1]. Due to the combination of low fertility and low mortality rates, the number and proportion of older adults has increased, and with them, long-term care and nursing home admissions as well [2]. Institutionalized older adults are characterized by a diminished intrinsic capacity (composite of physical and mental capacities) resulting in a vulnerable and fragile situation [3]. This scenario occurs because of the inexorable process of aging in conjunction with physical inactivity, which causes physiological changes such as loss of muscle mass, deterioration of neural mechanisms related to strength and power production, decrease in bone mineral density, and cognitive decline, among others [4]. All these alterations lead to reduced mobility, difficulty in performing activities of daily living (ADL), increased number of falls and hospitalizations, and an increased risk of mortality [5]. To address this negative situation, there is a need to develop and implement effective and sustainable strategies to improve older people’s quality of life and reduce public healthcare costs.

Exercise and nutritional supplementation constitute the best cost-benefit primary care interventions to delay and reverse frailty [6]. Exercise-based interventions can preserve and increase muscle mass, strength and power in institutionalized older adults, improving their quality of life, functionality and independency [[7], [8], [9]]. Specifically, the Vivifrail multicomponent exercise program has been shown to slow down or reverse the aging process in frail older adults with sarcopenia [10,11]. Nutritional strategies, such as HMB supplementation, play an anticatabolic role in skeletal muscle tissue, preserving muscle mass and function, and contributing to the relief of fatigue, muscular injury and inflammation [12]. The potential of HMB supplementation to enhance the effect of exercise training is attracting attention, but evidence remains inconclusive. Whereas it seems clear that HMB supplementation provides little or no extra benefits to exercise in trained athletes and healthy older adults [[13], [14], [15]], recent data in older adults with sarcopenia found that HMB significantly enhances the effect of resistance training on muscle strength, physical performance, muscle quality, and inflammatory factors [13,16,17]. These new results open the window to further examine the potential of HMB to complement exercise-based interventions in frail older adults with physical and muscular function limitations, such as institutionalized older people living in nursing homes [18].

Thus, this study aimed to investigate the synergist effect of multicomponent exercise training combined with HMB supplementation on disability, cognitive, physical and muscular function of institutionalized older people aged ≥ 70 years. Based on the previously identified positive effects for each of these isolated manipulations (multicomponent exercise and HMB supplementation), we hypothesize that the combination of both interventions in this population may produce a summative and/or superior positive effects.

## 2 Material and methods

### 2.1 Design and participants

A thorough description of the methodology of the present study has been reported elsewhere (NCT03827499) [19]. Briefly, this is a cluster-randomized, placebo-controlled study of 12 weeks of intervention and four parallel groups. Nursing homes were randomized to either of the four groups in clusters (i.e., a given group included participants from the same nursing home). The required sample size for each group was determined on the basis of the functional capacity, using the Short Physical Performance Battery (SPPB; 0–12) [20]. According to previous research on subjects with similar characteristics [21], a clinically relevant change is about 1.5 ± 1.0 points increments after 12 weeks. Differences of 2 points in total SPBB with a standard deviation of 3 points with a power of 80% and α of 0.05 can be estimated with 20 participants using the R software (v. 3.2.1) and the package sample size. Assuming a maximum loss of follow-up of 10%, we will recruit 22 adults ≥70 years per group. Nursing homes were then randomly assigned into one of the following four groups: exercise intervention plus placebo (EX), HMB supplementation only (HMB), exercise intervention plus HMB supplementation (EX + HMB), and control (CT) maintaining their usual routine in the nursing home. The inclusion criteria were: men and women aged ≥70 years old and living in a nursing home. The exclusion criteria were: having taken part in any intervention trial before; performing physical exercise regularly (at least 20 min/day and 3 days/week); having any HMB contraindication, intolerance or allergy; and having any pathological or metabolic condition incompatible with physical exercise.

### 2.2 HMB supplementation

The intervention groups with HMB supplementation (HMB and EX + HMB) received a 3-g daily dose of free acid HMB in powder form (MyProtein, Cheadle, Cheshire, UK) dissolved freely into 250 mL of water during the 12-week experimental period. This dose of HMB was chosen based on previous evidence [14,22]. The EX + HMB group were instructed to consume it after training sessions and at the same time on non-training days, whereas the HMB group ingested it consistently during their regular meal schedule. The EX group received stevioside as a placebo substance. Supplements were wrapped in indistinguishable envelopes and boxes, with an identification code for each participant and group. The supplementation and placebo intakes were monitored and ensured by medical staff working at the nursing home. An optimal nutritional and vitamin D status was ensured by nursing home medical staff.

### 2.3 Multicomponent exercise program

The exercise intervention groups (EX and EX + HMB) undertook an individualized multicomponent exercise training program (Vivifrail) for 12 weeks. The Vivifrail program is specifically designed for individuals aged 70 years and over, and it comprises six programs, referred to as "passports," which are tailored to each participant's functional status depending on their mobility limitations: serious (SPPB score of 0–3, Level A), moderate or frail (SPPB score of 4–6, Level B), slight or prefrail (SPPB score of 7–9, Level C), no limitation or robust (SPPB score of 10–12, Level D) and risk of falling (B + and C+) [23]. Individuals were categorized as having an elevated risk of falling if they fulfilled any of the following criteria: experiencing more than two falls within the past year, requiring more than 20 seconds in the Timed Up and Go test, exceeding 7.5 seconds in the 6-meter walking test, or having received a diagnosis of cognitive impairment [24]. The training program itself is centered around resistance (handgrip, biceps curl, squat and knee extension), balance (walking on toes and heels, around small obstacles, and stepping), flexibility (arm and hamstring stretching), and cardiovascular (walking) exercises. Participants with serious limitations and risk of falls (Level A, B+, C+) performed 5 days of multicomponent exercises and the remaining groups (Levels: B, C, and D) did 3 days of strength, balance and flexibility sessions, and 2 days of cardiovascular sessions. The duration of cardiovascular exercises varied, ranging from 3 min (Level A) to 20 min (Level D) with an intensity that facilitated participant conversation. Resistance exercises consisted of 3 sets of 12 repetitions, utilizing an absolute load (Kg) that allowed participants to complete a total of 30 repetitions (∼50% 1 repetition maximum [1RM]) [25]. Dynamic balance exercises were tailored to functional capacity, and all groups performed 3 sets of 10-second static stretching exercises for both upper and lower limbs. A standardized resting period of 2 min was conducted between all exercises and sets. All training sessions were conducted in-person by qualified sport scientists and supervised by a medical doctor, nurse and physiotherapist. The training program (SPPB-derived passport) was adjusted monthly to ensure optimal adaptations throughout the intervention. In addition, adherence to the training program was recorded, and participants who did not complete 80% of the sessions (48 of 60 sessions) were not considered for the analyses.

### 2.4 Outcomes

Anthropometric measures included body mass, height, and body mass index (BMI) using a portable scale and stadiometer (Seca 711, Hamburg, Germany).

Disability in basic and instrumental ADL were assessed through the Barthel index [26] and Lawton index [27], respectively. The Barthel questionnaire is composed of 10 items (feeding, bathing, dressing, grooming, bowels, bladder, toilet use, transfer, mobility and stairs) that result in a score ranging from 0 (total dependence) to 100 (complete independence). Conversely, the Lawton questionnaire assesses 8 items (using the telephone, shopping, cooking, household duties, laundry, use of transportation, responsibility for their medication and handling of economic matters) that result in a score ranging from 0 (total dependence) to 8 (total independence).

Cognitive function was evaluated using the mini-mental state examination (MMSE) questionnaire [28]. The MMSE questionnaire incorporates 11 items that measure attention and orientation, memory, registration, recall, calculation, language, and ability to draw a complex polygon, and results in scores ranging from 1 (lowest cognitive function) to 30 (highest cognitive function) points.

The SPPB [20] was used to assess physical function, comprising three tests: balance in three different positions (feet together, feet together but one of them more forward, and with one foot right in front of the other), habitual gait speed over a 4-m distance (4-m HGS), and 5-rep sit-to-stand (STS) performance. Specifically, the STS test was evaluated on a standardized chair (0.46 m height), and after the cue “ready, set, go!” the participants performed 5 STS repetitions as quickly as possible from the sitting position to the full standing position with the arms crossed over the chest. All tests were conducted twice with an adequate rest period in between (90−120 s) and strong verbal encouragement was provided. The best attempt was considered for further analysis.

Muscle function was measured by means of the power produced by the participants during the STS test using a validated equation [29]. Then, relative muscle power (i.e., normalized to body mass) and allometric muscle power (i.e., normalized to body size or height squared) were calculated accordingly [30]. For participants who were unable to complete 5 STS repetitions, muscle power was estimated using the equation provided elsewhere [31], which calculates the threshold of muscle power needed to perform 5 STS repetitions based on the participants’ anthropometric characteristics.

Handgrip strength was measured using a digital handheld dynamometer (Takei TKK 5401, Tokyo, Japan). The test was conducted with the participants seated in a chair with their arms fully extended along the trunk. Three attempts of 3-s maximum isometric contractions were performed with each hand, and the best attempt was considered for further analysis. Relative handgrip strength was calculated as absolute handgrip strength divided by body mass.

### 2.5 Statistical analysis

Data were presented as mean ± standard deviation or 95% confidence interval unless otherwise stated. The baseline characteristics of the participants were compared among intervention groups using linear mixed-effect models, in which subject allocation was introduced as a fixed effect, and subject ID as a random effect. Differences obtained by the study interventions were assessed by comparing changes (Post – Pre) provoked in the assessed outcomes using linear mixed-effect models. The assigned intervention was introduced as a fixed effect, subject ID as a random effect, and baseline values as a covariate. In all cases, the models were calculated considering maximum likelihood estimation and the best-fitting covariance structure. Furthermore, pairwise comparisons were carried out applying Bonferroni’s corrections. Cohen *d* effect sizes were calculated as the difference in group means divided by the standard deviation of the whole sample. Statistical analyses were conducted using SPSS (version 28.0, SPSS Inc., USA), and the level of significance was set at α = 0.05.

## 3 Results

**Fig. 1:**
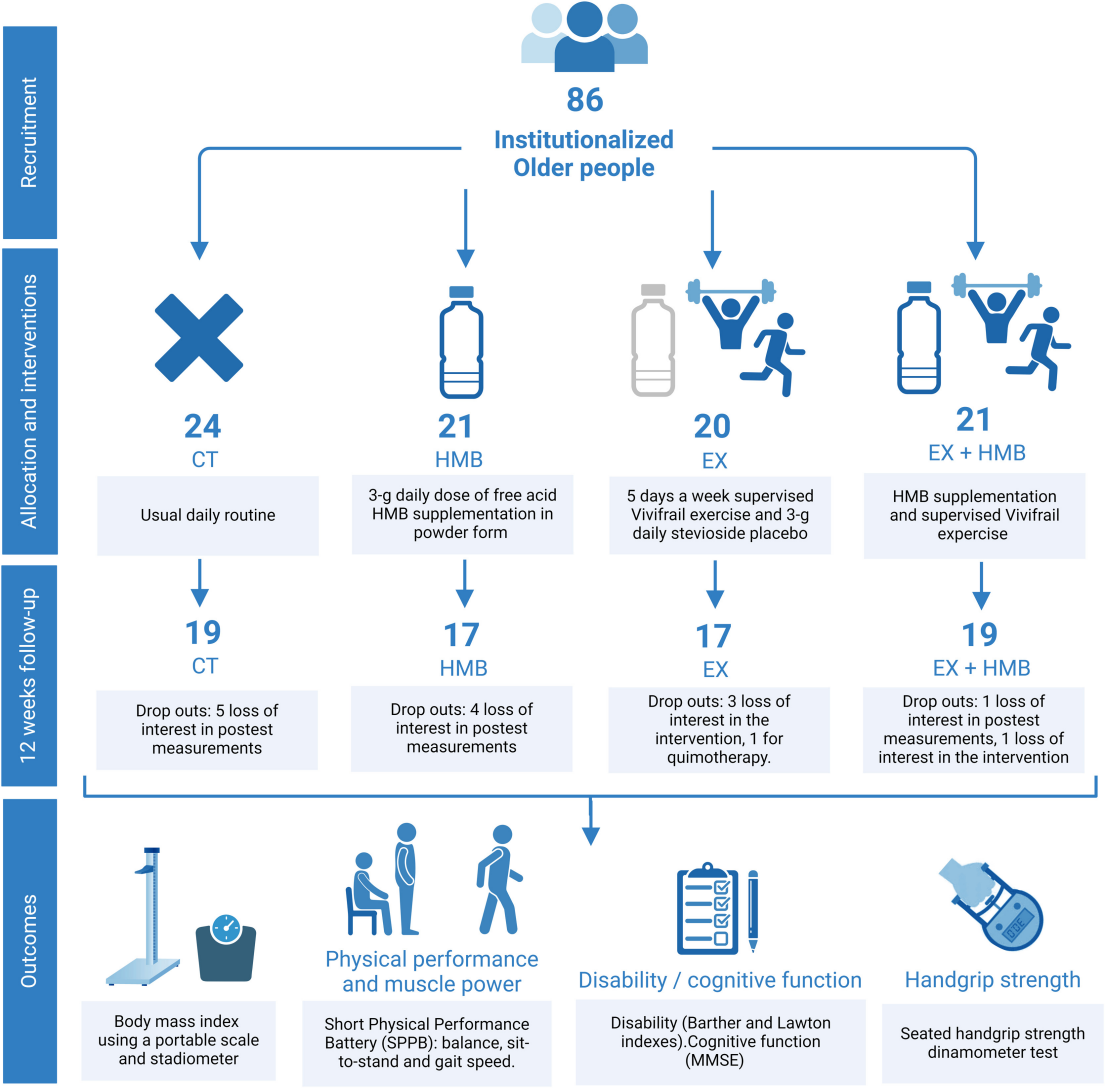
Schematic representation and timeline of study design.

Four nursing homes participated in the study, each one allocating one of the groups. A total of 86 nursing home residents entered the study (Fig. 1), of whom a total of 72 participants (62.5% women) completed the 12-week intervention and the post-test measurements: 19 CT (5 dropouts), 17 HMB (4 dropouts), 17 EX (3 dropouts) and 19 EX + HMB (2 dropouts). No adverse events were noted by the end of the intervention in any of the participants, and the attendance to the exercise sessions was 92.3 ± 7.2%. Baseline characteristics of the study participants are presented in Table 1. A group effect was noted for body mass, height, BMI and the Lawton index. Pairwise comparisons revealed differences in height between CT, HMB and EX + HMB (p = 0.005 and p = 0.002, respectively), in BMI between HMB and EX (p = 0.004), and in the Lawton index between CT and EX (p = 0.039).

**Table 1:** Baseline characteristics of the study participants.

Baseline characteristics	CT		HMB		EX		EX + HMB		Group effect
	Mean	SD	Mean	SD	Mean	SD	Mean	SD	
Sex (male/female)	5 / 14		7 / 10		5 / 12		10 / 9		
Age, years	86.2	8.9	85.8	9.5	78.2	9.8	82.2	10.7	0.056
Body mass, kg	59.7	16.4	62.7	15.2	72.8	20.3	73.2	17.8	**0.035**
Height, m	1.51	0.11	1.62^a^	0.11	1.56	0.10	1.63^a^	0.09	**<0.001**
BMI, kg·m^−2^	26.1	4.8	23.6	4.4	29.7^b^	5.6	27.6	5.2	**0.006**
MMSE score	18.4	8.8	21.6	4.9	20.4	6.2	21.0	6.5	0.481
Barthel index	79.5	17.2	82.4	19.1	82.1	20.2	80.9	22.6	0.969
Lawton index	2.3	2.0	3.2	1.9	4.5^a^	2.6	4.4	3.0	**0.019**
Handgrip strength, kg	17.0	10.9	16.1	5.5	20.0	8.8	17.5	9.6	0.594
Relative handgrip strength, kg·kg^−1^	0.27	0.11	0.26	0.07	0.27	0.09	0.24	0.10	0.644
SPPB score	6.9	2.8	5.7	2.9	6.5	3.8	6.1	3.4	0.709
4-m HGS, m·s^−1^	0.58	0.19	0.55	0.36	0.68	0.27	0.67	0.31	0.452
5-rep STS performance, s	13.31	5.84	19.40	6.94	15.80	5.90	18.57	7.32	0.057
Allometric STS power, W·m^−2^	46.9	27.3	34.9	9.4	55.4	24.7	49.7	26.1	0.067
Relative STS power, W·kg^−1^	1.80	0.90	1.50	0.44	1.90	0.70	1.80	0.74	0.457

Note. BMI, body mass index. CT, control group. EX, exercise group. HGS, habitual gait speed. HMB, β-hydroxy β-methylbutyrate. MMSE, mini-mental state examination. SD, standard deviation. SPPB, short physical performance battery. STS, sit to stand. Bold values indicate p < 0.05.

a Significantly different vs. control group (p < 0.05).

b Significantly different vs. HMB group (p < 0.05).

### 3.1 Anthropometric, cognitive function and disability

Changes in anthropometrics, cognitive function and disability in activities of daily living are shown in Table 2. A time effect was observed in cognitive function (i.e., MMSE score) (p < 0.001), whereas group effects were found regarding changes in body mass, cognitive function, and disability in instrumental ADL (i.e., Lawton index) (all p < 0.05). Within-group changes were observed in EX in terms of cognitive function (+2.9 points, p < 0.001), Barthel index (+8.1 points, p = 0.007) and Lawton index (+1.7 points, p < 0.001), and in EX + HMB in body mass (+1.6 kg, p = 0.003), BMI (+0.54 kg·m^−2^, p = 0.010) and cognitive function (+1.9 points, p = 0.002). No changes were found in CT or HMB (all p > 0.05), except in Lawton index (−1.1 points, p < 0.001 and −0.8 points, p = 0.012, respectively). In addition, differences were observed in changes in body mass between EX and EX + HMB (–0.84 ± 2.31 vs. 1.57 ± 2.32 kg, p = 0.013). Regarding changes in MMSE score, between-group differences were noted between the CT, EX and E + HMB groups (–1.13 ± 2.62 vs 2.91 ± 2.60 and 1.89 ± 2.60 points, p < 0.001 and p = 0.005, respectively). Finally, there were differences in the changes in the Lawton index between CT, EX and EX + HMB groups (–1.04 ± 1.18 vs. 1.67 ± 1.17 and 0.22 ± 1.16 points, p < 0.001 and p = 0.019, respectively), between HMB and EX (–0.75 ± 1.15 vs. 1.67 ± 1.17 points, p < 0.001), and between EX and EX + HMB (1.67 ± 1.17 vs. 0.22 ± 1.16 points, p = 0.003).

**Table 2:** Effects of the intervention in anthropometrics, cognitive function and disability in activities of daily living.

Outcome	Change^a^					Time effect	Group effect	Significantly different vs.
	Mean	95% CI	Δ%	ES	95% CI			
*Body mass,* kg								
CT	–0.05	–1.12 to 1.02	–0.09	0.00	–0.06 to 0.06	0.634	**0.016**	
HMB	–0.15	–1.26 to 0.96	–0.23	–0.01	–0.07 to 0.05			
EX	–0.84	–1.96 to 0.27	–1.16	–0.05	–0.11 to 0.02			EX + HMB
EX + HMB	**1.57**	**0.51 to 2.63**	**2.15**	**0.09**	**0.09 to 0.14**			EX
*BMI,* kg·m^−2^								
CT	0.13	–0.29 to 0.55	0.50	0.02	–0.05 to 0.10	0.507	0.059	
HMB	–0.15	–0.62 to 0.31	–0.64	–0.03	–0.11 to 0.06			
EX	–0.22	–0.69 to 0.24	–0.76	–0.04	–0.13 to 0.04			
EX + HMB	**0.54**	**0.12 to 0.97**	**1.97**	**0.10**	**0.02 to 0.18**			
*MMSE score*								
CT	–1.13	–2.37 to 0.10	–6.17	–0.17	–0.35 to 0.01	**<0.001**	**<0.001**	EX, EX + HMB
HMB	0.46	–0.90 to 1.81	2.14	0.07	–0.13 to 0.27			
EX	**2.91**	**1.66 to 4.17**	**14.32**	**0.43**	**0.25 to 0.62**			C
EX + HMB	**1.89**	**0.70 to 3.09**	**9.01**	**0.28**	**0.10 to 0.46**			C
*Barthel index*								
CT	–0.62	–6.52 to 5.27	–0.77	–0.03	–0.34 to 0.27	0.454	0.076	
HMB	–2.68	–9.13 to 3.78	–3.29	–0.14	–0.47 to 0.20			
EX	**8.13**	**2.06 to 14.19**	**9.90**	**0.42**	**0.11 to 0.73**			
EX + HMB	–0.11	–6.36 to 6.14	–0.14	–0.01	–0.33 to 0.32			
*Lawton index*								
CT	**–1.04**	**–1.60 to –0.48**	**–42.58**	**–0.42**	**–0.64 to –0.19**	0.864	**<0.001**	EX, EX + HMB
HMB	**–0.75**	**–1.34 to –0.16**	**–23.44**	**–0.30**	**–0.54 to –0.06**			EX
EX	**1.67**	**1.11 to 2.24**	**37.40**	**0.67**	**0.44 to 0.90**			CT, HMB, EX + HMB
EX + HMB	0.22	–0.36 to 0.80	5.05	0.09	–0.14 to 0.32			CT, EX

Note. BMI, body mass index. CT, control group. ES, effect size. EX, exercise group. EX + HMB, exercise plus HMB group. HMB, HMB group. MMSE, mini-mental state examination. 95%CI, 95% confidence interval. Bold values indicate p < 0.05.

a Changes were adjusted according to baseline values.

### 3.2 Physical function

Changes in physical performance are shown in Table 3. Time and group effects were observed for the 4-m HGS, 5-rep STS performance and SPPB score (all p < 0.001). Intra-group changes were observed in the EX and EX + HMB regarding 4-m HGS (+0.20 and +0.20 m·s^−1^, both p < 0.001), 5-rep STS performance (–4.91 and –3.75 s, both p < 0.001), and SPPB score (+2.9 and +2.4 points, both p < 0.001). No changes were found in CT or HMB groups (all p > 0.05). Concerning changes in 4-m HGS, between-group differences were detected between CT and EX + HMB (0.04 ± 0.17 vs. 0.20 ± 0.16 m·s^−1^, p = 0.043), and between HMB, EX and EX + HMB (–0.02 ± 0.16 vs. 0.20 ± 0.17 and 0.20 ± 0.16 m·s^−1^, p = 0.004 and p = 0.002, respectively). In addition, there were differences in the changes in 5-rep STS performance between the control group and EX (–0.42 ± 3.57 vs. –4.91 ± 3.33 s, p = 0.012), and between HMB, EX, EX + HMB (1.04 ± 3.34 vs. –4.91 ± 3.33 and –3.75 ± 3.47 s, p = 0.001 and p = 0.14, respectively). Finally, differences were found in changes in the SPPB score between CT, EX and EX + HMB (0.25 ± 1.85 vs. 2.97 ± 1.84 and 2.44 ± 1.84 points, p < 0.001 and p = 0.003), and between HMB, EX and EX + HMB groups (0.56 ± 1.85 vs 2.97 ± 1.84 and 2.44 ± 1.84 points, p = 0.002 and p = 0.018).

**Table 3:** Effects of the intervention in physical performance.

Outcome	Change^a^					Time effect	Group effect	Significantly different vs.
	Mean	95% CI	Δ%	ES	95% CI			
*4–m HGS, m/s*								
CT	0.04	–0.04 to 0.12	7.25	0.15	–0.12 to 0.42	**<0.001**	**<0.001**	EX + HMB
HMB	–0.02	–0.10 to 0.06	–3.48	–0.07	–0.35 to 0.21			EX, EX + HMB
EX	**0.20**	**0.11 to 0.28**	**28.89**	**0.69**	**0.38 to 0.99**			HMB
EX + HMB	**0.20**	**0.12 to 0.28**	**30.18**	**0.71**	**0.42 to 1.00**			CT, HMB
*5–rep STS performance, s*								
CT	–0.42	–2.50 to 1.65	–3.74	–0.06	–0.37 to 0.24	**<0.001**	**<0.001**	EX
HMB	1.04	–1.34 to 3.41	6.29	0.15	–0.20 to 0.50			EX, EX + HMB
EX	**–4.91**	**–6.70 to –3.12**	**–31.06**	**–0.73**	**–0.99 to –0.46**			CT, HMB
EX + HMB	**–3.75**	**–5.61 to –1.88**	**–20.18**	**–0.55**	**–0.83 to –0.28**			HMB
*SPPB score*								
CT	0.25	–0.60 to 1.09	3.58	0.08	–0.19 to 0.34	**<0.001**	**<0.001**	EX, EX + HMB
HMB	0.56	–0.34 to 1.45	9.75	0.17	–0.10 to 0.45			EX, EX + HMB
EX	**2.97**	**2.08 to 3.86**	**45.84**	**0.92**	**0.64 to 1.20**			CT, HMB
EX + HMB	**2.44**	**1.60 to 3.29**	**40.00**	**0.76**	**0.50 to 1.02**			CT, HMB

Note. CT, control group. ES, effect size. EX, exercise group. EX + HMB, exercise plus HMB group. HGS, habitual gait speed. HMB, HMB group. SPPB, short physical performance battery. STS, sit to stand. 95%CI, 95% confidence interval. Bold values indicate p < 0.05.

a Changes were adjusted according to baseline values.

### 3.3 Muscle power and handgrip strength

Changes in absolute and relative handgrip strength and allometric and relative STS muscle power are reported in Table 4. Time effects were observed only in the STS muscle power measures (both p < 0.001), whereas group effects were detected in both handgrip strength and STS power measures (all p < 0.05). No within-group changes were noted in absolute or relative handgrip strength among the participants (all p > 0.05), except for CT in relative handgrip strength (–0.03 kg·kg^−1^, p = 0.014). However, differences between groups were noted in changes in relative handgrip strength when comparing CT and EX (–0.03 ± 0.05 vs. 0.02 ± 0.05 kg·kg^−1^, p = 0.042). In terms of muscle power, within-group changes were observed in the EX and EX + HMB in both allometric STS power (+17.9 and +12.9 W·m^-2^, respectively, both p < 0.001) and relative STS power (+0.64 and 0.48 W·kg^−1^, respectively, both p < 0.001). Moreover, regarding changes in allometric STS power, there were differences between CT, EX and EX + HMB (–4.68 ± 12.33 vs. 17.9 ± 12.91 and 12.9 ± 12.38 W·m^-2^, both p < 0.001), and between HMB and EX (2.62 ± 11.84 vs. 17.9 ± 12.91 W·m^-2^, p = 0.003). Finally, there were differences in changes in relative STS power between the control group and EX and EX + HMB (–0.23 ± 0.51 vs. 0.64 ± 0.51 and 0.48 ± 0.51 W·kg^−1^, both p < 0.001), and between HMB and EX (0.11 ± 0.52 vs. 0.64 ± 0.51 W·kg^−1^, p = 0.023).

**Table 4:** Effects of the intervention on muscle strength and muscle power.

Outcome	Change^a^					Time effect	Group effect	Significantly different vs.
	Mean	95% CI	Δ%	ES	95% CI			
*Handgrip strength, kg*								
CT	–1.12	–2.40 to 0.17	–6.43	–0.12	–0.27 to 0.02	0.423	**0.037**	
HMB	–0.17	–1.50 to 1.16	–1.06	–0.02	–0.17 to 0.13			
EX	1.15	–0.18 to 2.49	5.77	0.13	–0.02 to 0.28			
EX + HMB	1.19	–0.06 to 2.45	6.83	0.13	–0.01 to 0.27			
*Relative handgrip strength, kg·kg^−1^*								
CT	**–0.03**	**–0.05 to –0.01**	**–10.00**	**–0.28**	**–0.52 to –0.05**	0.877	**0.026**	EX
HMB	0.01	–0.02 to 0.03	0.39	0.01	–0.24 to 0.26			
EX	0.02	–0.01 to 0.04	6.72	0.19	–0.06 to 0.43			CT
EX + HMB	0.02	–0.01 to 0.04	6.38	0.16	–0.08 to 0.39			
*Allometric STS power, W·m^−2^*								
CT	–4.68	–10.30 to 0.94	–9.96	–0.20	–0.43 to 0.04	**<0.001**	**<0.001**	EX, EX + HMB
HMB	2.62	–3.09 to 8.34	7.52	0.11	–0.13 to 0.35			EX
EX	**17.88**	**11.66 to 24.11**	**32.28**	**0.75**	**0.49 to 1.01**			CT, HMB
EX + HMB	**12.85**	**7.21 to 18.50**	**25.84**	**0.54**	**0.30 to 0.77**			CT
*Relative STS power, W·kg^−1^*								
CT	–0.23	–0.46 to 0.01	–12.86	–0.33	–0.66 to 0.01	**<0.001**	**<0.001**	EX, EX + HMB
HMB	0.11	–0.14 to 0.36	7.13	0.15	–0.20 to 0.51			EX
EX	**0.64**	**0.39 to 0.89**	**34.34**	**0.91**	**0.56 to 1.26**			CT, HMB
EX + HMB	**0.48**	**0.25 to 0.71**	**27.27**	**0.69**	**0.35 to 1.02**			CT

Note. CT, control group. ES, effect size. EX, exercise group. EX + HMB, exercise plus HMB group. HMB, HMB group. STS, sit to stand. 95%CI, 95% confidence interval. Bold values indicate p < 0.05.

a Changes were adjusted according to baseline values.

## 4 Discussion

As expected, the multicomponent exercise training was superior to HMB supplementation for improving disability in instrumental ADL, physical performance, and lower-limb muscle power. However, findings do not support the addition of HMB supplementation to enhance the benefits of multicomponent exercise in disability, and cognitive, physical and muscular function in institutionalized older people living in nursing homes. Accordingly, both interventions combined, however, had positive effects on disability in instrumental ADL, and cognitive, physical and muscular (power) function compared to controls, but not enough to improve disability in basic ADL or handgrip strength.

The number and proportion of older people living in nursing homes with some degree of dependency is expected to increase by 120% by the year 2050 [32]. Of note, older people living in nursing homes present higher levels of cognitive impairment, mobility limitations, frailty, disability, and hospitalization rate [33]. These negative conditions impose an important economic burden to public health systems [34,35], which makes the application of important countermeasures a public health priority [36].

In terms of cognitive impairments, previous studies conducted in rodents demonstrated that HMB supplementation preserved cognitive function [37,38], enhancing learning and working memory performance [37,39]. The ability of HMB supplementation to achieve these benefits in cognitive function would be partially explained by its ability to cross the blood-brain barrier [40]. In humans, there is only one previous study conducted on aviators that seems to align with these findings, reporting improvements in working memory, fluid intelligence, reaction time, and processing efficiency in young adults [41]. In the present study, HMB supplementation alone did not improve cognitive function assessed by the MMSE questionnaire in institutionalized older adults. However, a positive effect of exercise and exercise plus HMB supplementation was indicated by the reported 2.9 and 1.9 increases, respectively, in MMSE score. These increases are noteworthy, as both exceeded the minimum clinically important differences reported for people cognitively unimpaired (≥1.5 points) or those presenting mild cognitive impairment (≥1.7 points) [42]. Nevertheless, HMB supplementation did not augment the benefit already observed in the exercise plus placebo group, which questions the potential benefits of HMB over cognitive function in older people, at least in the short term. In contrast, the benefits of exercise training on cognitive function have been well documented in the literature [43,44], which has been mainly attributed to the release of myokines (e.g. brain-derived neurotrophic factor) into the bloodstream, and the ability of these myokines to cross the blood-brain barrier and stimulate neural plasticity [45].

Similarly, a recent systematic review and meta-analysis found that HMB supplementation combined with physical exercise yielded no or only marginal additional benefits in physical performance of older people compared to exercise alone [14]. However, no specific evidence existed on its effects on institutionalized older people, who present an increased prevalence of mobility limitations. In this sense, our study showed that only the exercise groups (alone or in combination with HMB) experienced benefits in disability in ADL, habitual gait speed, and STS performance. Particularly, improvements in SPPB score reached 3.0 and 2.4 points in the exercise alone and exercise plus HMB groups, respectively, which outperform the reported threshold for a clinically meaningful change (≥1 point) [46]. In contrast, no benefits resulted from HMB supplementation alone, nor did HMB supplementation increase the benefits derived from exercise when combined. This novel evidence extracted from institutionalized older people confirms the main findings provided by other studies [13,47,48] and the above-mentioned meta-analysis [14].

Regarding muscle function outcomes, HMB supplementation may improve muscle strength and power by promoting greater gains in skeletal muscle size [49]. Unfortunately, the present study did not aim to assess body composition and muscle size. However, the combination of exercise and HMB supplementation led to a significant increase in body mass when compared to changes in the exercise or HMB alone groups. Based on previous studies noting positive effects of HMB supplementation on muscle size [50], we may hypothesize that the participants in the exercise plus HMB group experienced an increase in muscle mass. Nevertheless, this hypothesis should be investigated in future studies conducted on nursing home residents. In any case, exercise alone attained significant increases in both allometric (+17.9 W·m^−2^) and relative muscle power (+0.64 W·kg^−1^), and the addition of HMB supplementation did not provide additional benefits (+12.9 W·m^−2^ and +0.48 W·kg^−1^, respectively). Importantly, these increments in muscle power were higher than the reported thresholds for being considered a minimal clinically important difference (e.g., in terms of relative STS power: ≥0.33 W·kg^-1^ in women and ≥0.42 W·kg^-1^ in men) [51]. These findings coincide with previous evidence highlighting the positive effects of exercise training on lower-limb muscle function independently from HMB supplementation [52]. On the other hand, a previous study that conducted a 12-week multicomponent exercise training intervention in institutionalized frail nonagenarians found improvements in maximal power at 30% 1RM (+96%) and 60% 1RM (116%) on bilateral leg press [53]. Other exercise training interventions including frail older people showed improvements in maximal power reaching 47% after a shorter period of time (6 weeks) [54]. These increases in muscle power are greater than those observed in our study (+25−35% in the exercise groups), which may be related to the fact that the other studies utilized specialized resistance training equipment and prescription [53,54]. In contrast, no significant increases in handgrip strength were noted in any of the study groups, as has been reported in other studies [13,48]. Only one previous study revealed significant enhancements in handgrip strength following HMB supplementation alone [16]. This aspect questions the ability of handgrip strength to monitor the effects of exercise or nutritional interventions on the functional trajectories of aging despite being one the most recommended assessment tools.

This research has certain limitations to be considered. Firstly, the use of self-reported scales in very old people may distort the reality. Secondly, although a comprehensive nutritional assessment by a registered dietitian was not conducted, the nursing home staff (including doctor and nurses) ensured an optimal diet. Thirdly, despite randomization, the exercise groups started with a lower self-reported disability (i.e., higher Lawton scores), but similar daily living functioning (Barther index) and functional capacity (SPPB values). Likewise, future studies should examine the potential benefits of higher but safe HMB doses or its combination with other nutritional supplements and the interaction with multicomponent exercise. Nonetheless, statistical analyses were adjusted to baseline levels to account for these initial differences. In terms of feasibility, the use of a tailored exercise program Vivifrail would allow us to conduct individualized exercise routines adapted to old people with or without disabilities. However, although we deliberately proposed a time-reduced measurement session, we noted that most dropouts reported a loss of interest in the follow-up without attrition to the intervention. Future studies examining very old adults should consider potential rejections to complete long-lasting testing sessions.

## 5 Conclusions

A multicomponent exercise program was effective to improve cognitive, physical and muscular function in institutionalized older adults, while HMB supplementation did not provide additional benefits when combined with exercise. These results emphasize the importance of physical exercise interventions in institutionalized, very old people as an essential basis for improving their overall health and quality of life. Conversely, the benefits of HMB as a unique intervention to increase functional and cognitive health are questioned. Additional studies are needed to identify specific changes in body composition caused by the combination of multicomponent exercise training and HMB supplementation.

## Funding

This work was supported by the Spanish Ministry of Science and Innovation (Grant PID2019-108202RA-I00funded by MCIN/AEI/ 10.13039/501100011033); by the Autonomous Community of the Region of Murcia, Regional Program for the Promotion of Scientific and Technical Research (Action Plan 2018), Seneca Foundation - Agency of Science and Technology, Region of Murcia (ID: 20872/PI/18); by CIBER – Consorcio Centro de Investigación Biomédica en Red – (CB16/10/00477), Instituto de Salud Carlos III, Ministerio de Ciencia e Innovación, and Unión Europea – European Regional Development Fund –; and by the Ministerio de Universidades of the Government of Spain (grant number FPU21/04717).

## Conflict of interests

The authors declare that they have no known competing financial interests or personal relationships that could have appeared to influence the work reported in this paper.
